# Frequency, risk factors, and outcome of neonatal meningitis in sepsis

**DOI:** 10.12669/pjms.40.9.8890

**Published:** 2024-10

**Authors:** Saeed Ahmed, Sundus Akhtar, Aysha Sultan, Ayaz ur Rehman

**Affiliations:** 1 Dr. Saeed Ahmed, FCPS Department of Pediatric Medicine and Child Health, Aga Khan University Hospital, Karachi, Pakistan; 2 Dr. Sundus Akhtar, FCPS Department of Pediatric Medicine and Child Health, Aga Khan University Hospital, Karachi, Pakistan; 3 Dr. Aysha Sultan, FCPS Department of Pediatric Medicine, National Institute of Child Health, Karachi, Pakistan; 4 Dr. Ayaz Ur Rehman, MBBS Department of Pediatric Medicine and Child Health, Aga Khan University Hospital, Karachi, Pakistan

**Keywords:** Neonatal meningitis, Sepsis, Complications

## Abstract

**Objective:**

To determine the frequency, associated risk factors, and outcome of meningitis in neonates presenting with sepsis at Aga khan Tertiary Care Hospital, Karachi.

**Methods:**

A descriptive cross-sectional study was conducted at pediatrics department of Aga Khan Tertiary University Hospital, Karachi, Pakistan from July 31, 2020, till January 30, 2021. Neonates of either gender admitted with neonatal sepsis were enrolled using non-probability consecutive sampling technique. Meningitis was diagnosed as per the findings of cerebrospinal fluid (CSF) along with the outcome in terms of death and early neurological complications such as subdural effusions and hydrocephalus.

**Results:**

Of 209 neonates with sepsis, meningitis was observed in 59 (28.2%) neonates. A significantly lower mean weight (p-value 0.024) while significantly higher mean duration of stay (p-value <0.001) was observed in patient with meningitis. Moreover, a significantly higher proportion of meningitis was observed in neonates who had fever (p-value 0.048), vomiting (p-value 0.009), abdominal distension (p-value <0.001), and blood culture positivity (p-value <0.001). Blood culture positive Methicillin-sensitive Staphylococcus aureus (MSSA) was considerably higher among neonates with meningitis. Of 59 neonates with meningitis, mortality was observed in 2 (3.4%) neonates. Positive CSF culture was observed in 6 (2.9%) while hydrocephalus was observed in 7 (11.9%) and effusion in 6 (10.2%) neonates.

**Conclusion:**

Neonatal meningitis is common in neonates presenting with sepsis, but mortality rate is low. Positive cultures, particularly with MSSA, further underscore the bacterial etiology in neonatal meningitis.

## INTRODUCTION

Neonatal sepsis represents a substantial contributor to both mortality and morbidity among neonates on a global scale.^1^,^2^ It is imperative to recognize that any neonate afflicted with neonatal sepsis is inherently susceptible to the development of meningitis.^2^ Neonatal meningitis is characterized as an infection affecting the meninges and central nervous system (CNS) within the first month of life.^3^ The incidence of bacterial meningitis in neonates ranges between 0.25 to one per 1000 live births and found in 25% of neonates with bacteremia.^4^ Up to 20% of newborns experience sepsis, with 7.7% succumbing to sepsis-related mortality.^5^

In a separate investigation, it was revealed that 57% of cases of gram-negative sepsis were concurrent with meningitis, while 36% of diagnosed cases of gram-positive sepsis were associated with meningitis.^6^ These findings highlight the burden of meningitis in neonates with sepsis. It is evident that an updated and comprehensive understanding of neonatal sepsis epidemiology, particularly in the context of local data, is crucial for addressing the burden of this disease. In developing country like Pakistan, neonatal sepsis and meningitis pose significant health challenges.^7^

This study was conducted at Aga Khan Tertiary Care Hospital in Karachi, Pakistan, with the aim of assessing the meningitis in neonates presenting with sepsis. Additionally, the study investigates clinical outcomes in neonatal meningitis cases, including hospital stay duration, early neurological complications, and mortality, to guide improved patient care and resource allocation.

## METHODS

This descriptive cross-sectional study was conducted at Pediatrics Department of Aga Khan Tertiary University Hospital, Karachi from July 31, 2020 till January 30, 2021.

### Ethical approval:

It was obtained from the ethical review committee of the institute prior conducting the study. (Ref. 2020-5305-14047 Dated September 07^th^ 2020). Moreover, signed informed consent was obtained from the parents/guardians of all study participants prior enrolment of the eligible study participants in the study.

### Inclusion & Exclusion Criteria:

All neonates of either gender diagnosed with sepsis were included. Whereas neonates with gross congenital neurological anomalies e.g., spina bifida, meningocele, and meningomyelocele were excluded. In addition, neonates with sepsis whose lumbar puncture did not perform were also excluded.

In this study, sepsis in neonates is operationally defined based on a combination of clinical and laboratory criteria. Clinically, neonates must exhibit two or more signs suggestive of systemic infection, such as temperature instability (fever > 38°C or hypothermia < 36°C), poor feeding, lethargy, irritability, respiratory distress (apnea, tachypnea, grunting), gastrointestinal symptoms (vomiting, diarrhea, abdominal distension), heart rate abnormalities (tachycardia > 180 beats/min or bradycardia < 100 beats/min), hypotension, poor peripheral perfusion, seizures, or a bulging fontanelle. Laboratory confirmation includes a positive blood culture for pathogenic bacteria, abnormal white blood cell count (leukocytosis > 25,000 cells/mm³ or leukopenia < 5,000 cells/mm³), elevated C-reactive protein (CRP > 10 mg/L) or procalcitonin levels (> 0.5 ng/mL), evidence of metabolic acidosis (pH < 7.35, bicarbonate < 20 mmol/L), and thrombocytopenia (platelet count < 150,000/mm³). Epi Info sample size calculator was used for the estimation of sample size taking the percentage of neonatal meningitis in neonates with sepsis 12.5%^8^ from the previous study conducted in a hospital in India resulted in the minimum sample size of 209 with a 5% margin of error and 97% confidence level.

In a newborn, meningitis was diagnosed when the cerebrospinal fluid (CSF) results met all the necessary requirements, including leucocyte count > 32 per mm3 and > 29 per mm3, glucose level 34 mg/dl and 24 mg/dl, and protein level > 170 mg/dl and > 150 mg/dl.^9^ The outcome was labelled in terms of mortality and length of stay. Moreover, complications such as hydrocephalus and subdural effusion were noted. This information along with the baseline characteristics of the patients including the age, gender, gestational age, and birth weight were collected.

### Statistical analysis:

Statistical Package for Social Sciences (SPSS) version 24 was used for the purpose of statistical analysis. Mean and standard deviation was calculated for quantitative variables like age, weight, and laboratory parameters. Frequency and percentages were calculated using gender, clinical features, meningitis, mortality status, and complications. The mean difference of quantitative variables with the presence of meningitis were explored using independent t-test whereas Chi-Square test/Fisher-Exact test was applied to see the association of meningitis with the qualitative predictor variables. The p-value of ≤0.05 was considered as significant.

## RESULTS

A total of 209 neonates with sepsis were included in the study. The mean age of the neonates was 9.36 ±7.63 days. There were 134 (64.1%) males and 75 (35.9%) were females. The mean gestational age was 35.21 ±3.92 weeks whereas the mean duration of stay was 13.47 ±11.44 days. Poor feeding was the most common clinical features observed in 64 (30.6%) of the neonates, followed by respiratory distress 58 (27.8%), seizures 55 (26.3%), lethargy 54 (25.8%), fever 45 (21.5%), excessive cry 28 (13.4%), vomiting 9 (4.3%), and abdominal distension 8 (3.8), and positive blood culture in 65 (31.1%) neonates (Table-I). Of these 65 blood culture positive neonates, the most common isolates organism was Klebsiella species, i.e., 14 (21.5%), followed by Acinetobacter 10 (15.4%), Methicillin-sensitive Staphylococcus aureus (MSSA) and Staphylococcus not aureus 9 (13.8%) neonates each, Group-B streptococcus (GBS) in 7 (10.8%) whereas 16 (24.6%) neonates had miscellaneous isolated organisms.

**Table-I:** Descriptive findings of the neonates with sepsis (n=209).

	n (%)
Age, days *(mean ±SD)*	9.36 ±7.63
Weight, kg *(mean ±SD)*	2.37 ±0.83
***Gender***	
Male	134 (64.1)
Female	75 (35.9)
Gestational age, weeks *(mean ±SD)*	35.21 ±3.92
Duration of stay, days *(mean ±SD)*	13.47 ±11.44
***Clinical Features***	
Fever	45 (21.5)
Lethargy	54 (25.8)
Poor Feeding	64 (30.6)
Excessive Cry	28 (13.4)
Vomiting	9 (4.3)
Seizures	55 (26.3)
Abdominal Distension	8 (3.8)
Respiratory Distress	58 (27.8)
***Examination Findings***	
Hb, g/dl *(mean ±SD)*	12.95 ±3.09
TLC, K/uL *(mean ±SD)*	13.47 ±8.43
Neutrophils, % *(mean ±SD)*	48.61 ±17.55
Lymphocytes,% *(mean ±SD)*	33.48 ±15.86
Platelets, K/uL *(mean ±SD)*	253.33 ±149.08
***Blood culture growth for organism***	
Positive	65 (31.1)
Negative	144 (68.9)

Hb: Hemoglobin, SD: Standard Deviation, TLC: Total Leucocyte Count.

**Fig.1:**
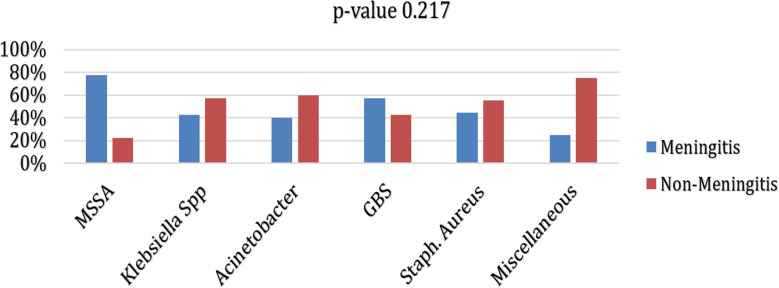
Comparison of organism isolates with meningitis among blood culture positive neonates (n=65).

The frequency of meningitis was observed in 59 (28.2%) neonates. Positive CSF culture was observed in 6 (2.9%) cases. A significantly lower mean weight (p-value 0.024) while significantly higher mean duration of stay (p-value <0.001) was observed in neonates with meningitis as compared to those without meningitis. Moreover, a significantly higher proportion of meningitis was observed in neonates who had fever (p-value 0.048), vomiting (p-value 0.009), abdominal distension (p-value <0.001), and CSF culture positivity (p-value <0.001), (Table-II). Although, an insignificant association of isolated organisms was observed with presence of meningitis (p-value 0.221), MSSA was found considerably higher among neonates who had positive blood culture. (Fig.1) Of 59 patients with meningitis, mortality was observed in 2 (3.4%) neonates. Whereas complications showed that hydrocephalus was observed in 7 (11.9%) and Subdural effusion in 6 (10.2%) neonates.

**Table-II:** Comparison of meningitis with predictor variables (n=209).

Variables	Meningitis		p-value

	Yes (n=59)	No (n=150)	
Age, days	10.17 ±8.11	9.05 ±7.44	0.340^α^
Weight, kg	2.16 ±0.82	2.45 ±0.81	0.024^α^
***Gender***			
Male	37 (62.7)	97 (64.7)	0.791^β^
Female	22 (37.3)	53 (35.3)	
Gestational age, weeks	34.42 ±4.09	35.51 ±3.83	0.071^α^
Duration of stay, days	18.41 ±14.50	11.53 ±9.34	<0.001^α^
***Clinical Features***			
Fever	18 (30.5)	27 (18.0)	0.048^β^
Lethargy	15 (25.4)	39 (26.0)	0.932^β^
Poor Feeding	17 (28.8)	47 (31.3)	0.722^β^
Excessive Cry	6 (10.2)	22 (14.7)	0.390^β^
Vomiting	6 (10.2)	3 (2.0)	0.009^β^
Seizures	11 (18.6)	44 (29.3)	0.114^β^
Abdominal Distension	7 (11.9)	1 (0.7)	<0.001^β^
Respiratory Distress	18 (30.5)	40 (26.7)	0.577^β^
Fontanelle	4 (6.8)	0 (0)	0.020^β^
***Examination Findings***			
Hb, g/dl	12.57 ±2.58	13.10 ±3.26	0.266^α^
TLC	13.57 ±7.42	13.44 ±8.82	0.918^α^
Neutrophils	47.19 ±17.98	49.17 ±17.41	0.464^α^
Lymphocytes	32.90 ±16.67	33.71 ±15.58	0.741^α^
Platelets	244.14 ±155.92	256.95 ±146.68	0.577^α^
***Blood culture***			
Positive	29 (44.6)	36 (55.4)	<0.001
Negative	30 (20.8)	114 (79.2)
CSF culture			
Positive	6 (100)	0 (0)	<0.001
Negative	53 (26.1)	150 (73.9)	

Hb: Hemoglobin, TLC: Total Leucocyte Count, kg: Kilogram,

α Independent t-test applied,

β Chi-Square/Fisher-Exact test applied, p-value ≤0.05 considered as significant.

## DISCUSSION

In the current study, the frequency of meningitis was observed in 28.2% neonates. This prevalence closely matched with the previously conducted international studies by Sarvepali et al. who reported meningitis in 29.9% neonates with sepsis^10^, Kaul et al 23%^11^, and Soni et al^12^ reported 33.5% meningitis in neonates with suspected sepsis referred to a pediatric emergency whereas Xu et al^13^ reported relatively higher (36.48%) bacterial meningitis in full-term neonates. Somewhat similar prevalence to the current study finding is reported in a study carried out in Lahore, Pakistan, i.e., 39.5%.^14^ The prevalence of meningitis found in our study, which is consistent with other international studies, indicates an early and urgent need to identify neonatal meningitis, necessitating screening in every case of sepsis and the development of preventative measures to avoid any fatal losses as a result.

According to the current study findings, hydrocephalus was observed in 11.9% and effusion in 10.2% of neonates with meningitis. Studies have reported that hydrocephalus occurs in approximately 7% of infants with neonatal meningitis whereas subdural effusion occurs in approximately 20-39 percent of neonates.^15^-^17^ In a research conducted in Iran, neurologic problems, such as hydrocephaly with and without abscess formation, subdural effusion, and brain edema, were seen in 20% of patients during brain sonography.^18^ Moreover, hydrocephalus was seen in two cases and CNS hemorrhage was seen 13 cases.^19^

As per the current study findings, poor feeding was the most common clinical features observed in 30.6% of the neonates, followed by respiratory distress 27.8%, seizures 26.3%, lethargy 25.8%, fever 21.5%, excessive cry 13.4%, vomiting 4.3%, and abdominal distension 3.8%. It is reported that early clinical features of neonatal meningitis are clinically unclear from those of sepsis.

In the current study, MSSA and Klebsiella species were considerably higher among neonates with meningitis. In a previous study, GBS and Escherichia coli were the leading pathogens in proven cases of neonatal meningitis.^13^

Mortality was found in two cases in the current study. Both cases had meningitis whereas isolated organisms were MSSA in one and Escherichia coli in the second neonate. Additionally, both hydrocephalous and effusion were observed in one case whereas no neurological complication was observed in the second case. Previous studies have reported that hydrocephalus and brain abscess can lead to long term morbidity and mortality in neonates.^20^,^21^

### Limitations:

The study was conducted at a single healthcare center. Moreover, the fact that the study was conducted in a hospital catering predominantly to patients with a privileged background, characterized by high socioeconomic status, raises concerns about the representativeness of the sample. Neonates from such backgrounds may have different risk factors and outcomes compared to those from less privileged backgrounds, thus limiting the applicability of the findings to a more diverse population. Additionally, the absence of therapeutic intervention data is a significant limitation as it prevents a comprehensive understanding of the management strategies employed and their impact on neonatal sepsis-associated meningitis.

### Strength of the study:

Despite these limitations, the study provides valuable baseline data on neonatal sepsis-associated meningitis, shedding light on its frequency, associated factors, and prognosis. This information can serve as a foundation for future research endeavors. To enhance the study’s impact, future research should consider expanding the sample size, incorporating multiple healthcare centers for a more diverse patient population, and including data on therapeutic interventions.

## CONCLUSION

In our study cohort, meningitis occurs in nearly one-third of neonates with sepsis. Significant clinical associations include lower neonatal weight, longer hospitalization, and symptoms such as fever, vomiting, and abdominal distension. Despite low mortality, the presence of MSSA in positive CSF cultures highlights the bacterial etiology of neonatal meningitis.

### Authors’ Contribution:

**SA** & **AR:** Concept, design, interpretation and analysis of data and final revision of manuscript. Responsible and accountable for the accuracy or integrity of the work.

**SU** & **AS:** Did data collection, Review and manuscript writing.

All authors have read and approved final version of manuscript.
